# Self-rated health supersedes patient satisfaction with service quality as a predictor of survival in prostate cancer

**DOI:** 10.1186/s12955-015-0334-1

**Published:** 2015-09-04

**Authors:** Digant Gupta, Kamal Patel, Christopher G. Lis

**Affiliations:** Cancer Treatment Centers of America® (CTCA), 500 Remington Road, Schaumburg, IL 60173 USA

## Abstract

**Background:**

We have previously reported that higher patient satisfaction (PS) with service quality is associated with favorable survival outcomes in a variety of cancers. However, we argued that patients with greater satisfaction might be the ones with better self-rated health (SRH), a recognized predictor of cancer survival. We therefore investigated whether SRH can supersede patient satisfaction as a predictor of survival in prostate cancer.

**Methods:**

Nine hundred seventeen prostate cancer treated at four Cancer Treatment Centers of America® hospitals between July 2011 and March 2013. PS was measured on a 7-point scale ranging from “completely dissatisfied” to “completely satisfied”. SRH was measured on a 7-point scale ranging from “very poor” to “excellent”. Both were dichotomized into two categories: top box response (7) versus all others (1–6). Patient survival was the primary end point. Cox regression was used to evaluate the association between PS and survival controlling for covariates.

**Results:**

The response rate for this study was 72 %. Majority of patients (*n* = 517) had stage II disease. Seven hundred eighty-seven (85.8 %) patients were “completely satisfied”. Three hundred nineteen (34.8 %) patients had “excellent” SRH. There was a weak but significant correlation between satisfaction and SRH (Kendall’s tau b = 0.18; *p* < 0.001). On univariate analysis, “completely satisfied” patients had a significantly lower risk of mortality (HR = 0.46; 95 % CI: 0.25-0.85; *p* = 0.01). Similarly, patients with “excellent” SRH had a significantly lower risk of mortality (HR = 0.25; 95 % CI: 0.11-0.58; *p* = 0.001). On multivariate analysis, SRH was found to be a significant predictor of survival (HR = 0.31; 95 % CI: 0.12-0.79; *p* = 0.01) while patient satisfaction was not (HR = 0.76; 95 % CI: 0.40-1.5; *p* = 0.40).

**Conclusions:**

SRH supersedes patient satisfaction with service quality as a predictor of survival in prostate cancer. SRH should be used as a control variable in analyses involving patient satisfaction as a predictor of clinical cancer outcomes.

## Background

Patient-reported outcomes such as patient satisfaction (PS) with service quality and self-rated health (SRH) are being increasingly used as important endpoints in cancer along with traditional endpoints of tumor response and survival. PS is an essential indicator of quality in health care and provides important information about the extent to which a patient’s needs and expectations are being met. It provides data concerning the quality of care and treatment delivered by physicians, paramedical staff and the hospital as a whole [1]. PS is becoming an increasingly important tool for providers to demonstrate patient focus and differentiation in the healthcare community, as well as enhance patient experience [2, 3]. The assessment of PS in an oncology setting is particularly salient where patients are subjected to increasingly complex treatments, exhaustive follow-ups, and numerous visits to hospital [4].

SRH, on the other hand, is a multidimensional construct that includes physical, social, psychological and functional domains at the very least. There is general agreement in the medical and scientific research community that patients are the best source of information regarding their health. Consequently, SRH assessment has become a valuable tool for both clinical practice and research [5]. SRH provides information about the impact of the disease and its treatment on multiple patient parameters that can aid physicians in selecting and managing antineoplastic and supportive therapy [5, 6].

There are extensive data in the literature demonstrating that pretreatment SRH can predict survival in several different types of cancers independent of the extent of the disease and other clinical prognostic factors [7–20]. More recently, we have reported that higher PS with service quality is associated with favorable survival outcomes in a variety of cancers including breast, colorectal and non-small cell lung [2, 3, 21]. However, while discussing our results, we cautioned the readers that patients with greater satisfaction with service quality might be the ones with better self-rated SRH, a well-established prognosticator of cancer survival. Concurrently, several recently published studies have indicated a possible link between SRH and PS in cancer [22–29].

Collectively, the above observations indicate that self-rated SRH might potentially confound the PS and survival relationship in cancer. However, to the best of our knowledge, no studies in the literature have explored this hypothesis in an oncologic setting. Understanding the interrelationships between SRH, PS and survival can have important implications in interpreting the results of studies that report on these measures.

The goal of this study, which is a sequel to our previously published research cited above, was to investigate if SRH is a potential confounder of the relationship between PS with service quality and survival in patients with prostate cancer undergoing treatment at a national network of oncology hospitals.

## Methods

### Study population

All prostate cancer patients who were seen in consultation at one of four Cancer Treatment Centers of America® (CTCA) hospitals between July 2011 and March 2013, who elected to have treatment at CTCA and who had not responded to a PS questionnaire within the preceding 60 days of treatment were eligible for this study. The four CTCA hospitals were CTCA Eastern, CTCA Midwestern, CTCA Southwestern and CTCA Western. The final surveyed cohort included a total of 917 patients. This study was approved by the Institutional Review Board (IRB) at CTCA. The need for informed consent was waived by the IRB because there was no direct patient contact in this study. This study involved collection of existing data from patient records in such a manner that subjects cannot be identified, directly or through identifiers linked to the subjects. Patient records/information was anonymized and de-identified prior to analysis.

### Questionnaire

The PS questionnaire used in this study was first implemented at our institution in August 2006. The instrument was developed based on input obtained from patient focus groups, and survey dimensions were collated from several existing studies or questionnaires of oncology patients [30–33]. This PS questionnaire covers the following dimensions: hospital operations and services, physicians and staff, and patient endorsements for others (friends and associates). The questionnaire was administered by trained survey associates at each CTCA hospital during a treating patient’s visit. Eligible patients were typically contacted while they were waiting for various appointments. The survey was paper-based and was completed by the patient and returned during that same visit at designated locations at each CTCA hospital. The survey was not anonymous because the survey data were linked with the electronic health records to create a comprehensive dataset which was anonymized and de-identified prior to analysis.

The questionnaire included PS items on: *team giving you the information you need to understand your medical condition*, *team explaining your treatment options, team involving you in decision making as much as you preferred, teams communicating with each other concerning your medical condition and treatment, care manager’s effectiveness in helping with your care when you are at home, team treating you with respect and in a professional manner, the response/call back from scheduling after you have left a message, waiting time for appointments* and *satisfaction with the treating medical oncologist (patient’s primary physician).* The questionnaire contained one overall PS item measured using the following question: “*considering everything, how satisfied are you with your overall experience with the institution*?” The questionnaire also contained one overall self-rated health (SRH) item measured using the following question: “*how would you rate your overall health during the last week?*” This questionnaire has not been validated previously.

### Statistical analysis

Patient survival was the primary end point, and was defined as the time interval between the date a patient first returned the patient survey and the date of patient’s death from any cause or the date of last contact/last known to be alive. The overall PS item was used as the primary independent variable in this study along with nine individual PS items. All PS items were measured on a 7-point scale ranging from “completely dissatisfied” to “completely satisfied.” The overall SRH item was used as the main study covariate/confounder. It was measured on a 7-point scale ranging from “very poor” to “excellent.” Because of skewed data distributions, both PS and SRH items were dichotomized into two categories for the purpose of this analysis: top box response (7) versus all others (1–6). Other control variables investigated for their relationship with survival were prior treatment history, stage at diagnosis, age and CTCA hospital. The prior treatment history variable categorized patients into those who had received definitive cancer treatment elsewhere before coming to CTCA and those who were newly diagnosed at CTCA. The stage at diagnosis variable was dichotomized into metastatic (stage IV) and non-metastatic disease (stages I-III). For CTCA hospital, dummy variables were created with CTCA Western as the reference category.

Univariate Cox proportional hazards models were used to determine which variables showed individual prognostic value for survival. Multivariate Cox proportional hazards models were then performed to evaluate the joint prognostic significance of all variables significant on univariate analysis. We used both block entry method (all variables entered together at the same time in one block) as well as the forward stepwise method. Forward stepwise method was used because, as is common in PS data, many of the individual items are highly correlated. Stepwise regression avoids the problem of multicollinearity because two highly correlated attributes will normally not both be entered in the model. Since ‘overall PS’ is highly correlated with other individual PS items, it was not included in multivariate Cox analyses when other PS items were used, in order to achieve model stability. Instead, “overall PS” was analyzed separately after adjusting for clinical and demographic factors. The effect of individual variables on patient survival was expressed as hazard ratios (HRs) with 95 % confidence intervals (CIs).

Cox regression with time-invariant covariates assumes that the ratio of hazards for any two groups remains constant in proportion over time. We checked this assumption by examining log-minus-log plots for categorical predictors. For continuous predictors, this assumption was checked using an extended Cox model with time-dependent covariates. Potential multicollinearity was assessed in two steps. Large values (>0.70) of Kendall’s tau b correlation coefficient were used as an initial screen for pairs of PS measures. Kendall’s tau b is an appropriate measure of association for categorical variables and is commonly used when both variables have the same number of categories. As a second check, the variance inflation factor (VIF) was used with the final model to verify that multicollinearity was not significantly influencing model coefficients [34, 35].

All data were analyzed using IBM SPSS version 23.0 (IBM, Armonk, NY, USA). A difference was considered to be statistically significant if the *p* value was less than or equal to 0.05.

## Results

### Response rate

A total of 1,274 returning prostate cancer patients were contacted at all four hospitals combined to participate in the survey between July 2011 and March 2013. However, only 917 patients responded. As a result, the response rate for this study was 72 %.

### Baseline patient characteristics

Table 1 displays baseline patient characteristics of the entire study population (*N* = 917). At the time of this analysis (May 2015), 57 (6.2 %) patients had expired. A majority of the patients were newly diagnosed at our institution and had stage II disease at diagnosis. Table 2 describes the distribution of PS items. Seven hundred eighty-seven (85.8 %) patients were “completely satisfied” with the overall service quality they received. The highest levels of dissatisfaction were observed for the following three individual PS items in terms of percent “not completely satisfied”: *waiting time for appointments* (21 %), *the response/call back from scheduling after you have left a message* (17.7 %) and *care manager’s effectiveness in helping with your care when you are at home* (17 %). Three hundred nineteen (35.8 %) patients had “excellent” SRH.

**Table 1:** Baseline patient characteristics

Variable	Categories	Number (Percent)
Age at the time of first survey	Mean	63
	Median	62.3
	Range	40.8-89.3
CTCA Hospital	Midwestern	426 (46.5)
	Southwestern	235 (25.6)
	Eastern	161 (17.6)
	Western	95 (10.4)
Stage at diagnosis	Stage I	127 (13.8)
	Stage II	537 (58.6)
	Stage III	107 (11.7)
	Stage IV	146 (15.9)
Treatment History	Newly Diagnosed	616 (67.2)
	Previously Treated	301 (32.8)

**Table 2:** Distribution of patient satisfaction items

How satisfied are you in the following areas:	Completely satisfied
Team giving you the information you need to understand your medical condition (*n* = 891)	761 (85.4)
Team explaining your treatment options (*n* = 881)	758 (86)
Team involving you in decision making as much as you preferred (*n* = 880)	776 (88.2)
Teams communicating with each other concerning your medical condition and treatment (*n* = 871)	741 (85.1)
Care manager’s effectiveness in helping with your care when you are at home (*n* = 731)	607 (83)
Team treating you with respect and in a professional manner (*n =* 884)	840 (95)
The response/call back from scheduling after you have left a message (*n* = 855)	704 (82.3)
Waiting time for appointments (*n* = 887)	701 (79)
Treating medical oncologist (*n* = 817)	736 (90.1)

• Items were dichotomized into two groups of “completely satisfied (7)” and “not completely satisfied (1–6)”

• Some sample sizes are less than 917 because of missing responses

### Correlation analysis

Table 3 displays Kendall’s tau b correlation coefficients among the PS items and SRH. The correlations among the PS items were weak to strong (ranging from 0.32 to 0.77) and all were statistically significant at the 0.01 level. The correlations between SRH and PS items were weak (ranging from 0.10 to 0.20) but statistically significant at the 0.01 level.

**Table 3:** Correlation analysis of patient satisfaction items with self-rated health

Kendall’s tau b	Overall satisfaction	Medical oncologist	Information	Explaining treatment	Involvement in decisions	Team communication	Help with home care	Respectful treatment	Scheduling	Waiting time	Overall health
Overall satisfaction	1.0										
Medical oncologist	.57	1.0									
Information	.52	.47	1.0								
Explaining treatment	.50	.49	.76	1.0							
Involvement in decisions	.49	.43	.67	.77	1.0						
Team communication	.53	.44	.73	.69	.70	1.0					
Help with home care	.44	.40	.57	.64	.58	.66	1.0				
Respectful treatment	.40	.36	.48	.48	.55	.50	.50	1.0			
Scheduling	.41	.36	.40	.40	.37	.40	.46	.33	1.0		
Waiting time	.41	.36	.38	.37	.36	.38	.45	.32	.62	1.0	
Overall health	.18	.19	.17	.19	.18	.14	.19	.10	.17	.20	1.0

• All correlations were significant at the 0.01 level

### Univariate analysis - predictors of patient survival

As shown in Table 4, the individual PS items that were significantly predictive of survival on univariate Cox regression analysis were: “*team giving you the information you need to understand your medical condition*”, “*team explaining your treatment options*”, “*team involving you in decision making as much as you preferred*”, “*teams communicating with each other concerning your medical condition and treatment*”, “*team treating you with respect and in a professional manner*”, and “*waiting time for appointments*”. In addition, the overall PS item was also significantly predictive of survival. Among the patient characteristics, SRH, prior treatment history, stage at diagnosis and age were significant predictors of survival.

**Table 4:** Univariate cox regression analysis

Variable	HR	95 % CI	*P*-value
Individual PS items			
Team giving you the information you need to understand your medical condition	0.45	0.24 to 0.82	0.009*
Team explaining your treatment options	0.40	0.22 to 0.73	0.003*
Team involving you in decision making as much as you preferred	0.31	0.17 to 0.56	<0.001*
Teams communicating with each other concerning your medical condition and treatment	0.37	0.21 to 0.66	0.001*
Care manager’s effectiveness in helping with your care when you are at home	0.55	0.29 to 1.06	0.08
Team treating you with respect and in a professional manner	0.39	0.17 to 0.92	0.03*
The response/call back from scheduling after you have left a message	0.75	0.37 to 1.5	0.41
Waiting time for appointments	0.51	0.29 to 0.90	0.02*
Treating medical oncologist	1.7	0.52 to 5.3	0.40
Overall PS item			
Overall patient satisfaction with the institution	0.46	0.25 to 0.85	0.01*
Patient characteristics			
Overall self-rated health (“not excellent” as referent)	0.25	0.11 to 0.58	0.001*
Treatment History (newly diagnosed as referent)	3.7	2.2 to 6.4	<0.001*
Stage at diagnosis (stages I-III as referent)	3.1	1.8 to 5.3	<0.001*
Age at first survey (used as a continuous variable)	1.1	1.02 to 1.1	0.002*
CTCA Hospital (overall effect)			0.06
Midwestern versus Western	2.1	0.49 to 9.0	0.32
Southwestern versus Western	4.2	0.98 to 18.2	0.06
Eastern versus Western	4.5	1.0 to 19.5	0.05*

• *P <0.05

• Individual and overall PS iems were dichotomized into two categories: “completely satisfied” (7) and “not completely satisfied” (1–6). “Not completely satisfied” was the referent group

• Self-rated health was dichotomized into two categories: “excellent” (7) and “not excellent” (1–6). “Not excellent” was the referent group

### Multivariate analysis - predictors of patient survival

Before proceeding with multivariate analysis, we checked the bivariate Kendall’s tau b correlation among the PS items in order to screen for observable multicollinearity. “*Team explaining your treatment options*” was highly correlated with two other PS items: “*team giving you the information you need to understand your medical condition*” (tau b = 0.76; *p* < 0.001) and “*team involving you in decision making as much as you preferred*” (tau b = 0.77; *p* < 0.001). As a result, “*team explaining your treatment options*” was not considered further in multivariate analysis. We also found a weak but significant correlation between overall PS and SRH (tau b = 0.18; *p* < 0.001).

**Fig. 1:**
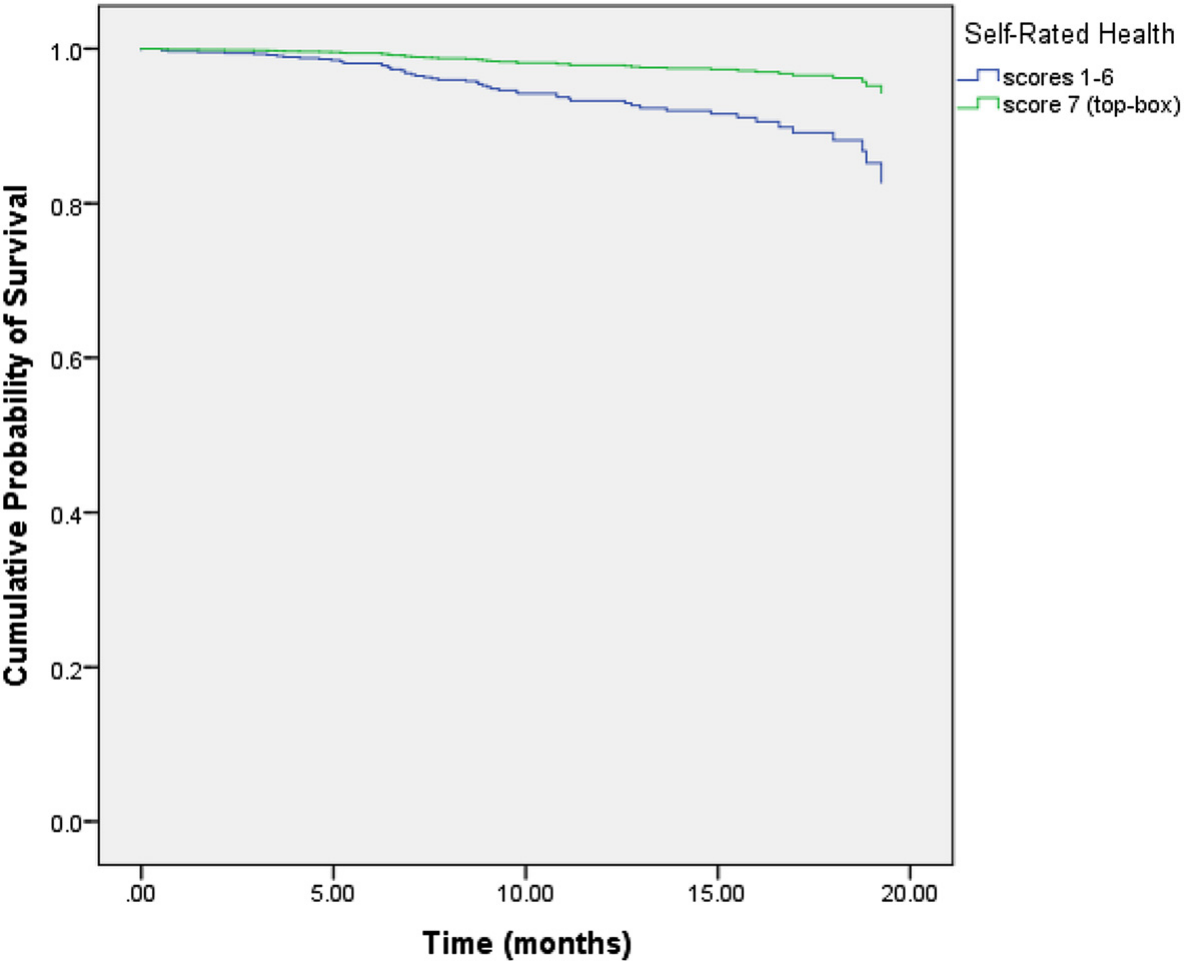
Adjusted survival curve for SRH. It displays the adjusted survival curves for the two categories of SRH after controlling for overall PS, stage at diagnosis, treatment history and age. The SRH curves were significantly different from each other (*p* = 0.01)

**Fig. 2:**
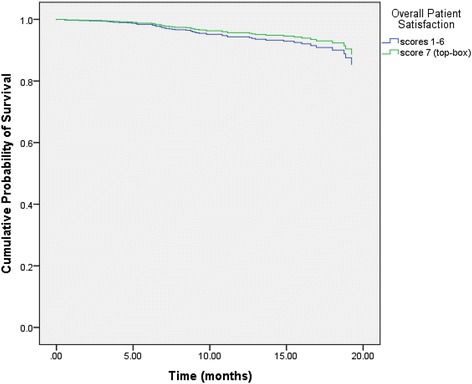
Adjusted survival curve for overall PS. It displays the adjusted survival curves for the two categories of overall PS after controlling for SRH, stage at diagnosis, treatment history and age. The PS curves were not significantly different from each other (*p* = 0.40)

Table 5 displays the results of the multivariate Cox regression for the following two models: “Model I” investigated five individual PS items controlling for SRH, stage at diagnosis, prior treatment history and age. “Model II” investigated the overall PS item controlling for SRH, stage at diagnosis, prior treatment history and age. In “Model I,” no PS item reached statistical significance whereas stage at diagnosis, treatment history, age and SRH were all found to be statistically significant. In “Model II,” the item pertaining to overall PS lost its statistical significance whereas SRH, stage at diagnosis, treatment history and age retained their statistical significance from univariate analysis. Figure 1 displays the adjusted survival curves for the two categories of SRH after controlling for overall PS, stage at diagnosis, treatment history and age. The SRH curves were significantly different from each other (*p* = 0.01). Figure 2 displays the adjusted survival curves for the two categories of overall PS after controlling for SRH, stage at diagnosis, treatment history and age. The PS curves were not significantly different from each other (*p* = 0.40).

**Table 5:** Multivariate cox regression analysis

Variable	HR	95 % CI	*P*-value
Model I: individual PS items			
Team giving you the information you need to understand your medical condition	0.87	0.30 to 2.5	0.80
Team involving you in decision making as much as you preferred	0.42	0.17 to 1.0	0.06
Teams communicating with each other concerning your medical condition and treatment	1.1	0.38 to 3.5	0.80
Team treating you with respect and in a professional manner	1.4	0.49 to 4.2	0.51
Waiting time for appointments	0.71	0.36 to 1.4	0.34
Overall self-rated health (“not excellent” as referent)	0.30	0.11 to 0.86	0.03*
Treatment History (newly diagnosed as referent)	2.9	1.6 to 5.2	<0.001*
Stage at diagnosis (stages I-III as referent)	3.4	1.8 to 6.2	<0.001*
Age at first survey (used as a continuous variable)	1.1	1.02 to 1.1	0.001*
Model II: overall PS item			
Overall patient satisfaction with the institution	0.76	0.40 to 1.5	0.40
Overall self-rated health (“not excellent” as referent)	0.31	0.12 to 0.79	0.01*
Treatment History (newly diagnosed as referent)	2.8	1.6 to 5.0	<0.001*
Stage at diagnosis (stages I-III as referent)	3.5	1.9 to 6.3	<0.001*
Age at first survey (used as a continuous variable)	1.1	1.02 to 1.1	0.002*

• *P <0.05

• Individual and overall PS items were dichotomized into two categories: “completely satisfied” (7) and “not completely satisfied” (1–6). “Not completely satisfied”    was the referent group

• Self-rated health was dichotomized into two categories: “excellent” (7) and “not excellent” (1–6). “Not excellent” was the referent group

• Model I investigates the individual PS items controlling for self-rated health, treatment history, stage at diagnosis and age

• Model II investigates the overall PS item controlling for self-rated health, treatment history, stage at diagnosis and age

The results of both models were confirmed using the forward stepwise approach. VIF values for the PS measures ranged from 1.2 to 2.3, none of which indicates a significant problem with multicollinearity [34, 35]. There was no evidence of non-proportional hazards in the multivariate models presented.

## Discussion

PS with service quality can at times be affected by resistance to the lifestyle changes that a cancer diagnosis and treatment entails and might not necessarily be a reflection of the patients’ perceptions of care with their healthcare providers. As a result, it becomes imperative to understand the relationship between PS and SRH, particularly within the context of cancer survival. In this study, we investigated the association between PS with service quality and survival after controlling for the effects of SRH in prostate cancer patients treated at a national oncology hospital network.

The univariate findings of this study suggest that patients completely satisfied with their service quality experience better survival outcomes compared to those who are not. However, after controlling for the effects of SRH in multivariate models, the relationship between PS and survival was rendered non-significant. On the other hand, SRH was found to be an independent predictor of survival in multivariate analysis after controlling for PS. This finding coupled with the observation that PS and SRH were significantly correlated (albeit weakly) suggests that SRH is a potential confounder of the relationship between PS with service quality and survival in prostate cancer. As a result, we propose that future studies involved in the collection and analysis of PS data should additionally collect information on SRH for more meaningful interpretation of their results. SRH should be an important stratification variable to consider when analyzing the data on PS in oncology.

Patients know better than anyone how they are feeling, and when patients report feeling less than in excellent health, this can be a sign that their disease is not responding well to treatment, or its associated side effects. Without clinical measures of treatment efficacy, these results also suggest that SRH is a reasonable proxy, since it has an independent effect on survival of the same order of magnitude as disease stage. This finding of a positive relationship between SRH and survival in oncology has been extensively reported in the literature over the last two decades [7–20]. Similarly, the finding of a positive relationship between PS and SRH has been recently reported in a few studies [22–29]. However, what is unique about this study is the fact that we have systematically and concurrently analyzed these inter-relationships in an oncology setting using survival as the primary endpoint.

Patient satisfaction, which is often assessed by heath care organizations, may be viewed as a useful, if imprecise, indicator of prognosis in prostate cancer patients, whether that association be due to improved general health, more positive emotions, or a combination of these. Although clinical indicators of prognosis are primary, these findings suggest that health care providers pay close attention to those patients who are less than completely satisfied during treatment. Doing so and alleviating any readily remedied causes of dissatisfaction may improve patient commitment to treatment protocols and secondary factors such as adequate nutrition.

There are some limitations of this study worth acknowledging. Our patient population was limited to only those patients who spoke English, so this study sample is, therefore, not broadly representative of prostate patients in general. Further, our study, which is exploratory by nature, used a non-validated questionnaire measuring PS and SRH. We were not able to control for patient co-morbidities due to lack of relevant data. Given that co-morbidities are significantly associated with patient survival, lack of adjustment for them leaves room for residual confounding in our analysis. Finally, we could not perform a comparison of baseline characteristics between responders and non-responders since we did not have any information available on non-responders. Since responders can differ from non-responders with regard to certain baseline demographic and clinical characteristics, the possibility of selection bias affecting our results cannot be ruled out.

The strengths of our study include a large sample size, a good response rate of 72 %, the fact that we measured PS and SRH as close to the time service was delivered as possible, and the fact that we used overall patient survival (the most objective and most commonly used health outcome measure in oncology) as our dependent variable. To the best of our knowledge, this study is the first in the health care literature to report on the association between PS with service quality and survival controlling for the confounding effects of SRH in a large sample of prostate patients.

## Conclusion

In conclusion, SRH confounds the PS-survival relationship in prostate cancer. SRH should be used as a control variable in analyses involving PS as a predictor of clinical cancer outcomes.
